# Efficacy of bezafibrate in two patients with mitochondrial trifunctional protein deficiency

**DOI:** 10.1016/j.ymgmr.2020.100610

**Published:** 2020-05-30

**Authors:** Tomonori Suyama, Masaru Shimura, Takuya Fushimi, Naomi Kuranobu, Keiko Ichimoto, Ayako Matsunaga, Masaki Takayanagi, Kei Murayama

**Affiliations:** Center for Medical Genetics, Department of Metabolism, Chiba Children's Hospital, 579-1 Heta-cho, Midori-ku, Chiba 266-0007, Japan

**Keywords:** Bezafibrate, TFP deficiency, Myalgia, Rhabdomyolysis, Fatty acid β-oxidation disorders (FAODs), l-carnitine, TFP, trifunctional protein, FAO, fatty acid β-oxidation, FAODs, fatty acid β-oxidation disorders, VLCAD, very-long-chain acyl-CoA dehydrogenase, LCHAD, long-chain 3-hydroxyacyl-CoA dehydrogenase, MCT, medium-chain triglycerides, CPT2, carnitine palmitoyltransferase II, CK, creatine kinase, AST, aspartate aminotransferase, ALT, alanine aminotransferase, CPA, cardiopulmonary arrest, QOL, quality of life

## Abstract

Mitochondrial trifunctional protein (TFP) deficiency is a rare inherited metabolic disorder caused by defects in fatty acid β-oxidation (FAO) of long-chain fatty acids, leading to impaired energy production. Fasting avoidance, fatty acid-restricted diets, and supplementation with medium-chain triglycerides are recommended as a treatment, but there are no pharmaceutical treatments available with strong evidence of efficacy. Bezafibrate, which enhances the transcription of FAO enzymes, is a promising therapeutic option for FAO disorders (FAODs). The effectiveness of bezafibrate for FAODs has been reported in some clinical trials, but few clinical studies have investigated its *in vivo* efficacy toward TFP deficiency.

Herein, we describe two Japanese patients with TFP deficiency. Patient 1 presented with recurrent myalgia since the age of 5 years. Laboratory findings showed increased serum levels of long-chain fatty acids and reduced expression of TFPα and TFPβ in his skin fibroblasts. Based on these findings, he was diagnosed with the myopathic type of TFP deficiency. Patient 2 suddenly exhibited cardiopulmonary arrest one day after birth. Elevated levels of creatine kinase and long-chain acylcarnitines were observed. Genetic analysis identified compound heterozygous variants in *HADHB* (c.1175C>T/c.1364T>G). He was diagnosed with the lethal type of TFP deficiency. Although both patients were treated with dietary therapy and l-carnitine supplementation, they experienced frequent myopathic attacks associated with respiratory infections and exercise. After the initiation of bezafibrate, their myopathic manifestations were markedly reduced, leading to an improvement in quality of life without any side effects.

Our clinical findings indicate that bezafibrate combined with other treatments such as dietary therapy may be effective in improving myopathic manifestations in TFP deficiency.

## 1 Introduction

Mitochondrial trifunctional protein (TFP) deficiency is a genetic disorder of the fatty acid β-oxidation (FAO) cycle [1]. TFP is located in the mitochondrial inner membrane and plays a role in long-chain FAO with very-long-chain acyl-CoA dehydrogenase (VLCAD) [2]. TFP is a multienzyme complex composed of four α subunits possessing long-chain enoyl-CoA hydratase and long-chain 3-hydroxyacyl-CoA dehydrogenase (LCHAD) activities (TFPα encoded by the *HADHA* gene) and four β subunits possessing long-chain 3-ketoacyl-CoA thiolase activity (TFPβ encoded by the *HADHB* gene) [2]. These three enzymes are impaired in general TFP deficiency, whereas only LCHAD activity is decreased in isolated LCHAD deficiency.

Similar to other FAO disorders (FAODs), decreased ATP production through the FAO cycle in TFP deficiency results in various clinical outcomes, especially during infection, exercise, diarrhea, and fasting [3]. TFP deficiency is classified into three types: (1) lethal type (neonatal-onset form), which includes the development of hypoglycemia, lactic acidosis, and cardiomyopathy during the neonatal period and is associated with high mortality; (2) intermediate type (infant-onset form), which is accompanied by hypoketotic hypoglycemia or hepatic dysfunction following infection and fasting; and (3) myopathic type (adult-onset form), which is characterized by muscle symptoms including intermittent myalgia or rhabdomyolysis [4]. In Japan, more than 10 patients with TFP deficiency have been reported to date, and the neonatal-onset form accounts for approximately half of these patients [4,5].

Regarding treatments, fasting avoidance, a diet restricted in long-chain fatty acids, and supplementation with medium-chain triglycerides (MCT) and essential long-chain fatty acids are recommended [6]. l-carnitine supplementation is believed to maintain the serum-carnitine concentration and eliminate toxic acylcarnitines [7,8], but its use is controversial due to lack of evidence of efficacy [6]. Bezafibrate, a commonly prescribed hypolipidemic drug, has shown promise as an FAOD treatment because it can enhance the transcription of β-oxidation enzymes [9,10]. Some clinical trials have reported the efficacy of bezafibrate in patients with VLCAD deficiency and carnitine palmitoyltransferase II (CPT2) deficiency [3,[11], [12], [13]]. However, there have been few studies on the *in vivo* effects of bezafibrate for TFP deficiency.

Herein, we describe two unrelated patients with TFP deficiency who showed a remarkable reduction in frequency of myopathic attacks with bezafibrate treatment.

## 2 Patient reports

### 2.1 Patient 1

**Fig. 1:**
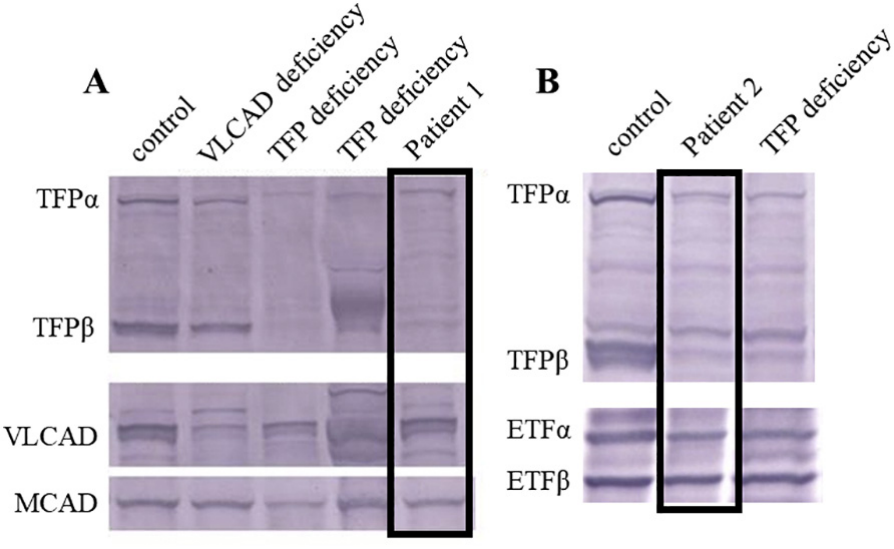
Western blot analysis of TFPα and TFPβ in patient fibroblasts. A. TFPα and TFPβ expression was decreased in patient 1 compared to their expression in control cells. B. Decreased expression of TFPα and TFPβ was observed in patient 2 compared to their expression in control cells. TFPα, α-subunit of TFP; TFPβ, β-subunit of TFP; VLCAD, very-long-chain acyl-CoA dehydrogenase; MCAD, medium-chain acyl-CoA dehydrogenase; ETFα, α-subunit of electron transfer flavoprotein; ETFβ, β-subunit of electron transfer flavoprotein; TFP, mitochondrial trifunctional protein.

A Japanese male infant was born as the first child to non-consanguineous parents, weighting 2502 g (−1.8 SD). His growth and development were normal. He had several episodes of severe myalgia and brown urine associated with exercise since the age of 5 years. At that age, blood tests showed elevated levels of creatine kinase (CK) and transaminases without hypoglycemia when he experienced muscle pain after swimming (CK, 14878 U/L; aspartate aminotransferase [AST], 328 U/L; alanine aminotransferase [ALT], 75 U/L) (Table 1). Serum acylcarnitine analysis using a critical sample showed increased levels of long-chain acylcarnitines (Table 2). Urine organic acid analysis revealed excessive excretion of 3-hydroxydicarboxylic acids. Causative variant was not identified in the coding regions of *HADHA* and *HADHB* by Sanger sequencing. Western blot analysis showed that TFPα and TFPβ expression was decreased in the patient's skin fibroblasts (Fig. 1A). He was diagnosed as having the myopathic type of TFP deficiency. His elder sister was also diagnosed with TFP deficiency at the same time.

**Table 1:** Laboratory findings.

	Patient 1 at myopathic attack	Patient 2 at CPA	Reference ranges
WBC (/μL)	5900	44,760	3900–9800
Hb (g/dL)	11.6	14.0	13.5–17.6
Plt (×10^4^/μL)	33.5	24.1	13.1–36.2
TP (g/dL)	7.1	3.8	5.7–7.3
Alb (g/dL)	4.6	2.4	4.0–5.0
AST (U/L)	328	514	15–50
ALT (U/L)	75	102	5.0–25
ALP (U/L)	1126	416	250–1600
LDH (U/L)	607	2855	200–800
CK (U/L)	14,878	41,730	180–430
BUN (mg/dL)	14	53.3	6.0–20
Cre (mg/dL)	0.38	1.46	0.2–1.3
UA (mg/dL)	5.2	15.9	1.8–7.5
Na (mmol/L)	139	142.1	135–147
K (mmol/L)	4.3	7.86	3.6–5.0
Cl (mmol/L)	105	104.2	98–108
NH_3_ (μg/dL)	17	63	30–80
Free carnitine (μmol/L)	44.3	28.7	36–74
Acylcarnitine (μmol/L)	14.2	10.0	6.0–23
pH	7.297	6.958	7.36–7.44
pCO₂ (mmHg)	38.1	83.1	36–44
Glu (mg/dL)	144	33	65–95
Lac (mg/dL)	18	13.5	5–12
BE (mmol/L)	−7.5	−15.4	−2.4–2.3
HCO₃^−^ (mmol/L)	18.0	18.2	22–26

WBC, white blood cell; Hb, hemoglobin; Plt, platelet; TP, total protein; Alb, albumin; AST, aspartate aminotransferase; ALT, alanine aminotransferase; ALP; alkaline phosphatase; LDH, lactate dehydrogenase; CK, creatine kinase; BUN, blood urea nitrogen; Cre, creatinine; UA, uric acid; Glu, glucose; Lac, lactate; BE, base excess; CPA, cardiopulmonary arrest.

**Table 2:** Acylcarnitine profile in critical samples.

	Patient 1	Patient 2	
	Serum (Reference range)	Dried blood spots at 5 days old (Reference range)	Serum at 20 days old (Reference range)
C0 (μmol/L)	43.5 (31.3 ± 8.4)	30.05 (10.0–60.0)	20.95 (10.0–55.0)
C2 (μmol/L)	7.3 (6.2 ± 2.1)	22.44 (5.0–45.0)	16.97 (4.0–60.0)
C14:1 (μmol/L)	0.66 (0.08 ± 0.04)	**1.59** (<0.4)	**0.46** (<0.1)
C14-OH (μmol/L)	NA	NA	**0.17** (<0.1)
C16 (μmol/L)	**0.46** (0.09 ± 0.04)	**8.7** (0.6–7.0)	0.36 (<0.5)
C18 (μmol/L)	**0.29** (0.04 ± 0.02)	2.16 (0.15–2.1)	0.085 (<0.3)
C18:1 (μmol/L)	**0.40** (0.11 ± 0.05)	2.91 (0.3–3.2)	0.29(<0.46)
C16-OH (μmol/L)	**0.102** (0.005 ± 0.001)	NA	0.11(<0.8)
C16:1-OH (μmol/L)	NA	**0.38** (<0.1)	NA
C18-OH (μmol/L)	NA	**0.57** (<0.1)	**0.053** (<0.05)
C18:1-OH (μmol/L)	**0.093** (0.005 ± 0.001)	**0.64** (<0.07)	0.12 (<0.7)

NA, not available. Abnormal values are in bold.

As his intermittent myalgia and rhabdomyolysis recurred every 2 or 3 months, l-carnitine supplementation and dietary therapy consisting of restricted long-chain fatty acid consumption and MCT substitution were initiated. Nevertheless, his myalgia was not ameliorated. As he grew and his exercise level increased, the frequency of myopathic attacks and outpatient visits increased. His elder sister who was also treated with diet therapy and carnitine supplementation had an uneventful course.

**Fig. 2:**
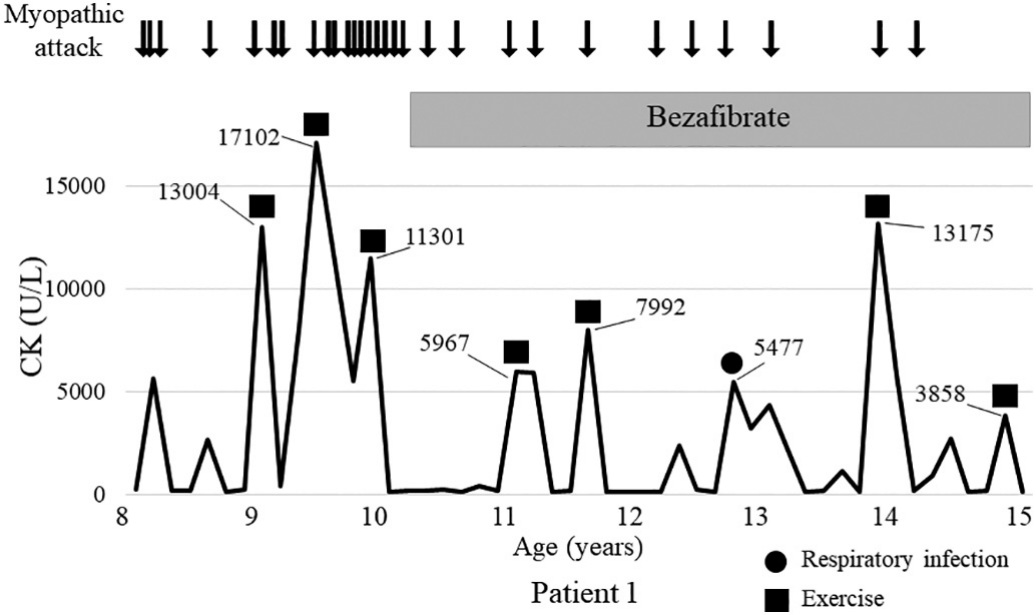
Clinical course of patient 1 before and after initiation of bezafibrate. Bezafibrate was initiated at the age of 10 years. The frequency of myopathic attack and peak levels of serum creatine kinase (CK) were reduced by treatment.

Bezafibrate was initiated at the age of 10 years (Fig. 2). The dose of bezafibrate was 10 mg/kg/day for the first week and was increased to 20 mg/kg/day on the second week. The frequency of exercise-induced myalgia decreased from 10 times to 3 times per year, and his outpatient visits became less frequent leading to an improvement in his quality of life (QOL). At the time of writing this report, he was 18 years old and bezafibrate side effects had not occurred. Retinopathy and cardiomyopathy did not develop.

### 2.2 Patient 2

A boy was born at 38 weeks of gestational age by cesarean section, as the second child of non-consanguineous Japanese parents. His birth weight and height were 2848 g (−0.2 SD) and 50.0 cm (1.0 SD), respectively. He suddenly presented with cardiopulmonary arrest (CPA) on day 1 after birth, while he was being held in his mother's arms. Cardiopulmonary resuscitation for 50 min restored spontaneous circulation. Laboratory tests showed mixed respiratory and metabolic acidosis with hypoglycemia (glucose, 33 mg/dL). CK, AST, and ALT levels were elevated (CK, 41730 U/L; AST, 514 U/L; ALT, 102 U/L) (Table 1). Acylcarnitine analysis showed elevated levels of long-chain acylcarnitines(Table 2), and urine organic acid analysis detected increased excretion of 3-hydroxydicarboxylic acids. Western blot analysis showed decreased expression of TFPα and TFPβ (Fig. 1B). Sanger sequencing identified compound heterozygous variants in *HADHB* (NM_000183;c.1175C>T:p.Ala392Val/c.1364T>G:p.Val455Gly). Both variants had been identified in previous studies [5,14]. He was diagnosed as having the lethal type of TFP deficiency. Laboratory analysis at 2 years old showed hypocalcemia (calcium, 6.0 mg/dL [8.7–10.2 mg/dL reference range]), hyperphosphatemia (inorganic phosphorus, 9.0 mg/dL [3.5–6.0 mg/dL]), and a reduced parathyroid hormone level (intact parathyroid hormone, 8.0 mg/dL [10–65 mg/dL]). He was diagnosed with hypoparathyroidism, and oral alfacalcidol was initiated.

**Fig. 3:**
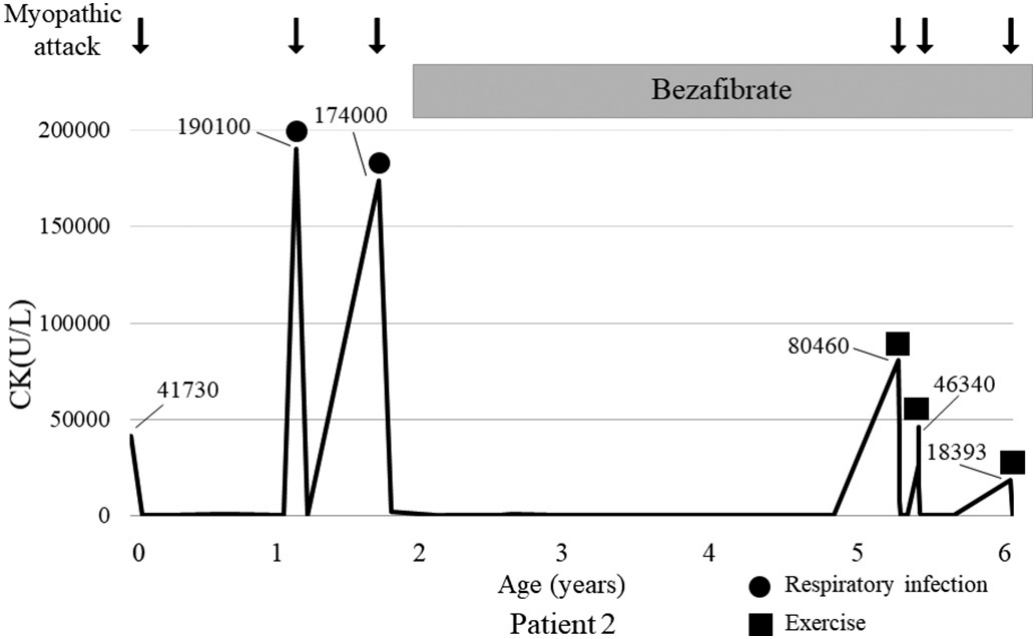
Clinical course of patient 2 before and after initiation of bezafibrate. Bezafibrate was started at the age of 2 years and myopathic attack was completely prevented for 3 years. However, rhabdomyolysis developed again at the age of 5 years. Therefore, exercise restriction was initiated. Myopathic attack did not develop again from 6 years old onward. CK, creatine kinase.

After the CPA episode, he was treated with dietary therapy including long-chain fatty acid restriction, MCT milk, and l-carnitine supplementation, but he experienced two episodes of rhabdomyolysis induced by infection. In the first episode, he required intensive care including mechanical ventilation. Therefore, bezafibrate was initiated at 2 years old to prevent future bouts of rhabdomyolysis (Fig. 3). The dose regimen was the same as that used for patient 1. After the initiation of bezafibrate, rhabdomyolysis did not appear for 3 years, but developed again as the patient grew and increased his activity. An exercise restriction was added and myopathic attacks including rhabdomyolysis had not occurred up to the date of writing this report, when the patient was 8 years old. Both of these patients have been mentioned in a previous study [4].

## 3 Discussion

To the best of our knowledge, this is the first detailed report describing a remarkable reduction in the frequency of myopathic attacks after bezafibrate initiation in two patients with TFP deficiency. Both patients had been treated with l-carnitine supplementation and dietary therapy, but myopathic attacks could not be prevented. Bezafibrate administration successfully reduced the myopathic attacks and improved patient QOL even in the lethal type of TFP deficiency.

Bezafibrate is an agonist of the peroxisome proliferator-activated receptor that plays a key role in the transcriptional control of genes encoding mitochondrial FAO enzymes [15]. The efficacy of bezafibrate for FAODs including carnitine-acylcarnitine translocase deficiency, VLCAD deficiency, and CPT2 deficiency has been reported in *in vitro* studies and case reports [8,[16], [17], [18], [19]]. An open-label, non-randomized trial of bezafibrate in six patients with CPT2 deficiency was first performed by a French group. That study revealed that bezafibrate improved physical activity and myopathic manifestations, suggesting its therapeutic efficacy in the muscle form of CPT2 deficiency [11,12]. Furthermore, an open-label clinical trial for VLCAD deficiency and CPT2 deficiency conducted in Japan showed that bezafibrate can improve the QOL of patients with FAODs, although the frequency of myopathic attacks and levels of CK and serum acylcarnitines were not changed [3,13]. In contrast, a Danish group reported that bezafibrate is ineffective in improving clinical symptoms in CPT2 and VLCAD deficiency [20]. Thus, bezafibrate has remained controversial as a clinical treatment for FAODs; further studies are required to elucidate the effectiveness of this drug.

To date, a clinical trial of bezafibrate for TFP deficiency has not been performed. Djouadi et al. demonstrated that bezafibrate improved FAO capacities in fibroblasts from six (23%) of 26 TFP-deficient patients [10]. A previous study indicated that three of five patients with TFP deficiency showed clinical improvement with bezafibrate treatment [4]. In the current study, we provided the detailed clinical courses and effectiveness of bezafibrate treatment in two of these patients with positive outcomes. Bezafibrate in combination with l-carnitine supplementation and dietary therapy could reduce the frequency of myopathic attacks and decrease the peak levels of CK during these attacks, leading to an improvement in QOL, although exercise restriction was also required in patient 2.

Although causative variants were not identified in the coding regions of *HADHA* and *HADHB* in patient 1, reduced expression of TFPα and TFPβ by immunoblotting guided the diagnosis of TFP deficiency. The patient could have unidentified variants, such as deep intronic variants, that alter gene expression; thus, RNA sequencing or cDNA analysis may be necessary to identify the causative variants. Patient 2 harbored reported variants in *HADHB* (c.1175C>T/c.1364T>G). It has been reported that TFP-deficient patients who have the c.1175C>T in at least one allele are predisposed to develop hypoparathyroidism in the Japanese population [4]. Accordingly, patient 2 presented with hypoparathyroidism at the age of 2 years and was treated with alfacalcidol.

In conclusion, our findings suggest that bezafibrate combined with other treatments such as dietary therapy is effective in improving muscle manifestations in TFP deficiency. Going forward, further studies are needed to elucidate the efficacy of bezafibrate for treating TFP deficiency.

## Ethics approval

This is an observational retrospective patient report that did not involve any research-based patient intervention. All interventions were intended to diagnose and treat the patient. No aspect of the case report is in contradiction with the Helsinki Declaration of 1975, as revised in 2000.

## Submission declaration and verification

This report has not been published previously and is not under consideration for publication elsewhere. Publication of this report is approved by all authors.

## Funding

This research did not receive any specific grant from funding agencies in the public, commercial, or not-for-profit sectors.

## Patient consent

Written informed consent for the present study was obtained from the patients' parents. The consent form was approved by the ethical committee of Chiba Children's Hospital.

## Authors' contributions

T.S., A.M, and M.S. conceptualized and wrote the report; T.F., K.I., N.K., and M.T. were involved in data curation. M.S. and K.M critically reviewed and edited the draft.

## Declaration of Competing Interest

The authors declare no conflict of interest.
